# Reducing Geographic Barriers for Breast Cancer Diagnosis

**DOI:** 10.1200/GO.22.55000

**Published:** 2022-05-05

**Authors:** Emily Adaniya, Manisha Bhatia, Stephen Kiptoo, Lucy Najala Wabende, Nicholas Kisilu, Silvanus Kibiwot, Sally Jepkirui, Dorice Adhiambo Awuor, Patrick Loehrer, Joanna Hunter-Squires, Naftali Busakhala

**Affiliations:** ^1^ Indiana University, School of Medicine, Indianapolis, IN; ^2^ Indiana University, Bloomington, IN; ^3^ Academic Model Providing Access to Healthcare—Moi Teaching and Referral Hospital, Eldoret, Kenya; ^4^ Moi University, School of Medicine, Eldoret, Kenya

## Abstract

Increasing travel times to the nearest diagnostic facilities and cancer centers are correlated with decreasing prevalence of breast cancer diagnosis and increasing stage of cancer at diagnosis. Moi Teaching and Referral Hospital (MTRH), in Uasin Gishu County, houses the only public cancer center in western Kenya. A MTRH program, the Academic Model Providing Access to Healthcare Breast and Cervical Cancer Control Program (ABCCCP) hosted health fair breast cancer screening events and mentored nurses at local health facilities to complete clinical breast exams (CBEs). The objective is to understand the effect of the ABCCCP in reducing geographic barriers for breast cancer screening/early diagnosis.

**METHODS:**

This is a retrospective review of the 61,392 patients who underwent breast cancer screening at a ministry of health facility in western Kenya from 2017-2021. ABCCCP hosted health fairs targeted at communities and mentored nurses at health facilities to complete clinical breast exams (CBEs) to target individual patients. Facility distances from MTRH were mapped via Google Maps. Descriptive analyses were performed to a significance of α < 0.05.

**RESULTS:**

Two-thirds of the screening/early diagnosis CBEs occurred in five of the 22 counties in western Kenya: Busia, Uasin Gishu, Trans-Nzoia, Bungoma, and Kakamega. However, these five counties only had 25% of all ABCCCP screening sites in western Kenya. Only Uasin Gishu and Kakamega provided chemotherapy while Busia and Trans-Nzoia had additional programming promoting preventative healthcare through integration of community health volunteers. In these five counties, no association between the distance of the ministry of health facilities from MTRH and number of CBEs completed existed.

**CONCLUSION:**

ABCCCP programming reduced some geographic disparities in breast cancer screening/diagnosis in the top five counties. It may be result of the number of mentored nurses and health fair events. Further work should be completed to understand if a relationship between geographic distance from MTRH and the stage and treatment patterns of these patients is also reduced by ABCCCP.

